# Human rights education in social studies in the Netherlands: A case study textbook analysis

**DOI:** 10.1007/s11125-018-9431-3

**Published:** 2018-03-12

**Authors:** Frauke de Kort

**Affiliations:** 1 Netherlands Institute for Human Rights, P.O. Box 16001, 3500 DA Utrecht, The Netherlands; 2 University of Twente, Drienerlolaan 5, 7522 NB Enschede, The Netherlands

**Keywords:** Human rights education, Citizenship education, Social studies, Textbook analysis, Netherlands

## Abstract

Citizenship education is one of the main aims of the mandated subject of social studies in secondary schools in the Netherlands. Moreover, the learning outcomes of social studies refer to constitutional rights. Internationally, citizenship education and human rights education are considered to be mutually reinforcing. One may, thus, expect that Dutch school textbooks include elements of human rights education. This article presents the analysis of a popular social studies textbook in the Netherlands, applying a number of human rights education criteria. The study shows that basic information on human rights is lacking, despite ample opportunity to integrate such themes. Most worrisome is the conveyance of potential misinformation about human rights due to the chosen formulation of rights-related issues. This can, in part, be traced back to the textbook authors’ (mis)understanding of human rights.

## Introduction

The state of the Netherlands is bound to respect, protect, realize, and fulfill a broad range of human rights through its ratification of international human rights treaties (Netherlands Institute for Human Rights 2015; Oomen and Vrolijk 2010). Furthermore, the Netherlands, like many other countries, has committed itself to promoting human rights education (Council of Europe 2010; United Nations 2011). The call to “put promise into practice” and give human rights education a solid place in formal education has increased in the Netherlands in recent years. (See, for instance, Netherlands Institute for Human Rights 2015; Oomen and Vrolijk 2010; Platform Mensenrechteneducatie 2008; Platform Onderwijs2032 2016; SLO 2012, 2014). To date, however, human rights education has no official place in the curriculum of the Dutch schooling system.

In international and national policy documents (Council of Europe 2010; Education Council 2012; Platform Onderwijs2032 2016; SLO 2012; United Nations 2004), and equally in academic literature (Bron and Thijs 2011; Fritzsche 2007; Osler 2016; Tibbitts and Fritzsche 2006), human rights education is frequently connected to citizenship education. The UN World Programme on Human Rights Education, launched in 2005, considers human rights education a prerequisite for democratic citizenship, as education should “enable all persons to participate effectively in a free and democratic society governed by the rule of law” (United Nations 2004, para. 3 [d]). More recently, the Charter on Democratic Citizenship and Human Rights Education of the Council of Europe (2010) describes the link between the two as follows:

Education for democratic citizenship and human rights education are closely inter-related and mutually supportive. They differ in focus and scope rather than in goals and practices. Education for democratic citizenship focuses primarily on democratic rights and responsibilities and active participation, in relation to the civic, political, social, economic, legal and cultural spheres of society, while human rights education is concerned with the broader spectrum of human rights and fundamental freedoms in every aspect of people’s lives. (Section I, para 3)

Since 2006, schools in the Netherlands have been under the legal obligation to contribute to “active citizenship and social integration” (Education Inspectorate 2006, p. 13). One area of concern of citizenship education, according to the inspection framework for this law, is “basic values, and democracy and the rule of law”. Thus, schools are required to offer their learners ways to acquire knowledge, attitudes, and skills needed to participate as a citizen in a democratic society under the rule of law, including knowledge of Dutch and European polity (Education Inspectorate 2006).

Schools are free to exercise this task in the manner they deem most appropriate, which we must consider in light of the Dutch education system. This system is characterized by its so-called freedom of education, laid down as such in the Constitution. For primary education (ages 4–12) and lower secondary education (ages 12–14), learning outcomes describe the knowledge and skills that its learners should attain. However, schools (and teachers) are free to choose how to achieve these outcomes, which methodologies or textbooks to use, and even which outcomes to emphasize over others. To a large extent, schools in upper secondary education (ages 14–18) enjoy the same freedom. At this level, official (national) exam programs mandate learning outcomes. However, the students take national standardized exams only in the last year of secondary education. The results of these exams, in combination with earlier school-based test results, make up students’ final grades for secondary school. We find the roots of this freedom of education in political struggles over the financing of religion-based schools in the past—which still echo today.

The Education Inspectorate—a division of the Ministry of Education responsible for inspection and review of the educational quality of individual schools, educational institutions, and the education system as a whole—oversees the implementation of the 2006 law on active citizenship and social integration. In its most recent reports on the state of education (Education Inspectorate 2016 and 2017) the Education Inspectorate noted that, in practice, schools rarely pay systematic attention to citizenship education. Concrete learning outcomes are often lacking or not evaluated by the school. The Education Council, an independent governmental advisory body that advises the minister, parliament, and local authorities—has also called upon the state to provide clarity about the core content of the citizenship education task for schools (Education Council 2012).

Despite the freedom in education and lack of guidance on the content of citizenship education, one subject has citizenship education as one of its two main functions (Olgers, Van Otterdijk, Ruijs, De Kievid, and Meijs 2014b). The subject of *maatschappijleer* (translated here as “social studies”) is mandatory for all students in upper secondary school and thus offered in a systematic manner with concrete learning outcomes. In other countries, human rights education is often integrated into subjects such as social studies (Meyer, Bromley, and Ramirez 2009; Tibbitts 2002). Thus, a study of human rights education in the Dutch context naturally leads us to the school subject of social studies.

Here, I present the results of a study on human rights as taught in social studies classes in the Netherlands, based on my content analysis of a school textbook used at secondary schools for teaching social studies, *Seneca Maatschappijleer havo-vwo* (Olgers, Schra, and Veldman 2014a), and interviews with the textbook authors. In this mixed-methods analysis, I looked at the quantity and quality of references to human rights and the rationales the authors presented for their choices.

I chose this textbook for several reasons. First, it was recently published (2014) and developed at a time when human rights education was put on the Dutch political agenda, as I noted at the beginning of this article. Thus, we might expect to see links between human rights education and citizenship education. For instance, in the same period, Platform Mensenrechteneducatie [Human Rights Education Platform] — a partnership of various NGOs for human rights education— conducted research (2008), and the SLO (2012), upon the request of the Ministry of Education, developed a ‘curriculum’ for citizenship education and human rights education. Secondly, this textbook has a chapter entitled “Human Rights Dilemmas”, indicating that it contains some treatment of human rights. Finally, two social studies teachers and a university teacher-trainer for social studies developed the textbook. The university teacher-trainer was coauthor of the *Handboek Vakdidactiek Maatschappijleer* [Handbook social studies pedagogy] (Olgers et al. 2014b), which forms the core literature for any (student) social studies teacher in the Netherlands. The authors had found the existing social studies textbooks wanting in developing critical thinking around political dilemmas and fundamental values.

A school textbook is not the only factor determining what happens in classrooms. In general, and in the Dutch situation in particular, one should not underestimate the role of schools and teachers. However, indications are that textbooks do have a considerable influence in education (Druba 2006; Pingel 2010). Specifically for the Netherlands, the Netherlands Institute for Curriculum Development, SLO, indicated in its “Learning plan for fundamental education of the future” (SLO 2014) that the theoretical freedom that schools have to make their own content choices is, in reality, limited by a strong orientation towards textbooks. The SLO speaks of a special kind of “self-imposed prescription” and notes that teachers often lack the capacity to make solid learning plans to fully use their professional freedom (SLO 2014). Therefore, I focused on the intended curriculum of one of the social studies textbooks, assuming that—since social studies textbooks in the Dutch context are an important instrument in education—many teachers look to school textbooks for some guidance in their teaching as well as for classroom exercises and test materials.

I first explain how I developed the criteria for my textbook analysis, referencing the learning goals of the exam program in social studies in the Netherlands. After laying out the methodology of my study, I present the results of both the content analysis and the author interviews. I conclude with a summary and several recommendations.

## Literature review

In preparation for the textbook analysis, I conducted a literature review to develop an analytical framework for studying the degree and kind of human rights content in social studies textbooks. This review included international policy documents on human rights education, recent scholarship on human rights education, textbook studies on human rights in textbooks, and policies of the Dutch social studies curriculum.

I found the most recent definition of “human rights education” in the United Nations (UN) Declaration on Human Rights Education and Training (2011). This definition entails three important aspects of human rights education, which are also found in academic literature: education *about* human rights, *through* human rights, and *for* human rights (Bajaj 2011; Fritzsche 2005; Oomen 2009; Tibbitts 2002). Like citizenship education, human rights education is a broad concept ranging from knowledge and skills to attitude and action (Council of Europe 2010; Fritzsche 2005).

Over the years, academics have developed various theories and models about human rights education (see Bajaj 2011 for an overview). For the present research, I relied on the categories of human rights education that Felisa Tibbitts (2002) developed, primarily because I could most easily apply these to formal education in a Western context like the Netherlands (school textbook analyses in Germany equally refer to the Tibbitts model; see Lenhart 2006). Other categories tend to be drawn from and focus on more development contexts or a very specific aim beyond the scope of citizenship education in the Netherlands—such as human rights education for global citizenship, human rights education for coexistence, or human rights education for transformative action (see Bajaj 2011). To operationalize the concept of human rights education for this research, I selected Tibbitts’ Value and Awareness model, which is based on a philosophical-historical approach to human rights. This model is applicable to human rights education in formal education, which, most importantly, aims to realize a basic knowledge and understanding of human rights among the learners. It is less about skills and transformative action (Tibbitts 2002). Consistent with the model, I here examine the content—that is, the knowledge—aspect of human rights education, and not subject-related pedagogy or the pedagogy of human rights education.

In terms of human rights knowledge goals, I considered three models for the development of the content criteria of human rights: the 10 categories developed by the Georg Eckert Institute for International Textbook Research (GEI) (Druba 2006; Lenhart 2006; Weinbrenner and Fritzsche 1998), Weinbrenner’s “didactic cube of human rights” (Weinbrenner and Fritzsche 1998), and a list of the knowledge components of human rights education based on research in human rights education in the Netherlands (Oomen and Vrolijk 2010). Upon studying many school textbook analyses, Weinbrenner and Fritzsche (1998) concludes that human rights are often only implicitly mentioned. He thus proposes that the core content of human rights education be the human rights themselves; that is, with an emphasis on their normative character. Thereafter, human rights can be described in their historical perspective and the reality of human rights discussed through political and social questions. This approach seemed very suitable for the subject of social studies in the Netherlands, which addresses political and social questions.

After identifying the general content requirements of human rights education, I studied these in relation to the content requirements of the 2016 exam program of social studies (for the Dutch secondary-school levels *havo* and *vwo*) and the functions and aims of social studies as per the leading teacher-training manual, *Handboek Vakdidactiek Maatschappijleer* (Olgers et al. 2014b). From the study of the exam program, it was clear that human rights was not an explicit learning goal of the subject of social studies. However, from the perspective of the formulation of certain learning outcomes and the aims of social studies according to leading academics in the field (Olgers et al. 2014b), many possibilities for human rights to be included emerged.

The exam program requirements were mainly aimed at knowledge in four domains: rule of law, parliamentary democracy, the welfare state, and multicultural society. (Note that in Dutch it is called “pluriform society”, as, in current political discourse, the “multicultural” society has failed due to the alleged lack of integration of various cultures.) Some of the learning outcomes in each of these domains did mention rights – freedom rights (fundamental freedoms), political rights, and social rights—but only in relation to the Constitution, not to human rights. From a human rights perspective, not only would the normative character of the Dutch Constitution be important but also the binding character of the human rights treaties that the Netherlands had ratified. At the same time, the exam program required each learner to be able to describe the reality of and tensions in the rule of law, parliamentary democracy, the welfare state, and multicultural society. This related to the three aims of the subject of social studies according to Olgers et al. (2014b), namely: (1) political and social literacy, (2) political and social judgement, and (3) the ability for political and social participation. This fits perfectly with the historical perspective, the reality of human rights, and the political and social questions Weinbrenner and Fritzsche (1998) mentions, thus affirming the viability of a human rights content-focused approach to the textbook.

## Methodology

In alignment with the literature review, I developed a framework for the human rights content analysis, which consisted of four main categories: introduction to human rights, protection mechanisms, specific human rights, and a category called “human rights in practice”. I further subdivided these main categories and associated them with keywords that could be applied to the content analysis. The literature review had revealed the basic general content for knowledge on human rights. Subsequently, I adapted this content for relevance to the Netherlands (for instance, excluding human rights documents of regions other than Europe and including relevant European ones) and to the subject of social studies (choosing those rights that relate to the constitutional rights, domains, and dilemmas mentioned in the exam program). I intended the category “introduction to human rights” to cover only the very basic notion of human rights: a definition, the underlying values, their normative character, and the most relevant examples of treaties. Likewise, I chose the category “protection mechanisms” as a separate category in line with the exam program domain of the rule of law; and the category “specific human rights” and its concepts as being in line with the focus on constitutional rights, on the one hand, and other human rights relevant to the exam program domains of “welfare state” and “multicultural society”, on the other. I put non-discrimination and interrelatedness as subcategories here, as they pertained to the concepts of “inequality” and “inequity” in relation to specific rights. Lastly, I chose the category of “human rights practice” to go into more detail regarding the historical and present contexts, which related to the learning outcomes of historical developments of democracy, rule of law, the welfare state, and multicultural society as well as current challenges and political dilemmas. Table 1 below overviews these elements.

**Table 1:** Categories

Main category	Subcategory	Concept
A. Human rights: general	Human rights: explanation	Word/ explanation
	Principles and underlying values	Principles of human dignity, universality, and inalienability Underlying values of freedom, equality, and respect
	Normative character of human rights (treaties and declarations)	Human rights are binding Duties and obligations of states Self determination
		International UDHR ICCPR ICESCR CEDAW CRC CERD CRPD Geneva Conventions Rome Statute on the International Criminal Court
		Europe European Convention on the Protection of Human Rights and Fundamental Freedoms European Social Charter Charter of the Fundamental Rights of the European Union
B. Protection mechanisms	National	Constitution Democratic rule of law National human rights institution
	European	European Court of Human Rights Court of Justice of the European Union
	International: treaty bodies, complaint mechanisms, reporting procedures, UN-agencies and organization	Treaty bodies UN Security Council UNICEF OHCHR UNHCHR ILO International Criminal Court
	NGOs	Examples Amnesty International, Human Rights Watch, Netherlands Committee of Jurists, Defense for Children
C. Specific human rights	Civil and political rights	Right to life Prohibition of torture, cruel, inhuman or degrading treatment or punishment Prohibition of slavery Right to liberty and security of person Right to fair trial and legal assistance Right to privacy Right to freedom of thought, conscience and religion Freedom of expression Prohibition of propaganda for war and advocacy of national, racial or religious hatred that constitutes incitement to discrimination, hostility or violence Right to peaceful assembly Right to freedom of association (including trade unions) Right to marry and to form a family Right to a name and nationality Right to vote and participation Equality before the law Right of minorities to their own culture, religion and language
	Economic, social and cultural rights	Economic rights Right to work and favorable conditions of work Right to and of trade unions
		Social rights Right to social security, including social insurance Right to social assistance Right to decent standard of living Right to health Right to education
		Cultural rights Right to cultural life, science and intellectual property
	Non-discrimination	
	Indivisibility and interrelatedness	
D. Human rights in practice	History of human rights – 3 generations	History and development of human rights
	Criticism on human rights	Criticism on the universality of human rights Criticism on certain human rights
	Dilemmas and clashing human rights	Absolute rights Limitations grounds Dilemmas Clashing rights
	Human rights violations	Genocide Crimes against humanity Violation of individual rights

In my content analysis of the textbook, I considered the above criteria in answering the following questions: To what extent do human rights feature in the textbook? If they do feature, is the content correct? Are there important omissions or missed opportunities?

I disregarded the illustrations and focused on text only, which, in this textbook, consisted of authors’ text, including exercises, but also some text excerpted from other sources with the exercises. I used a combination of quantitative and qualitative content analysis methods.

The first step was a frequency analysis of the word ‘human rights’ to get an initial impression of the presence of human rights in this book. I then employed a frequency analysis of the selected human rights concepts, which focused not only on the literal occurrence of the word but also on the presence of the concept as content; that is, whether the book implicitly or explicitly featured the concept. Next, I coded those passages to indicate whether the concept was present in the text authored by the writers, in text quoted from another source, or in an exercise. As a third step, I revisited the coded passages to determine whether the book discussed the concepts explicitly in their relationship with human rights and thus would contribute to an increased understanding (knowledge) of human rights, or not—and scored accordingly. Fourthly, I carefully reread the coded passages that I had scored as “explicit” to see whether the content was correct from a human rights perspective. Lastly, I analyzed the text on important omissions in content and “missed opportunities”, which mainly concerned the passages scored “implicit”.

In an attempt to interpret the findings of the content analysis and look for obstacles and opportunities to include human rights education in the school subject of social studies, I also included a content analysis of interviews with the textbook authors. My assumption was that the authors’ knowledge and attitude towards human rights would influence the extent to which human rights education featured in the textbook. As proxies for knowledge, I chose their academic background and specialization as well as their subjective theory (Flick 2006) of human rights. Their subjective theory would indicate their explicit and implicit assumptions about human rights and human rights education, which knowingly or unknowingly could have influenced the way the textbook presented human rights. I used semi-structured interviews to collect the data for this content analysis (Silverman 2010). A few open questions formed the basis of the interviews. The questions centred on knowledge of human rights and human rights education, the connection between human rights education and social studies, opportunities and obstacles for adapting the textbook. My aim was to let the authors talk as freely as possible and analyze their answers afterward.

The face-to-face interviews took place on two separate days, each interview lasting approximately two hours, and I recorded them in their entirety for analysis. During the first interview with the two teacher-authors and the first half of the second interview with the pedagogue-author, I played the naïve interviewer (Flick 2006; Hermanns 2010) to elicit—as a student—as many insights as possible from the experts. Towards the end of the second interview with the more experienced pedagogue-author, I was able to share some preliminary findings and observations from the textbook analysis to get his opinion on these. Then, I coded the transcribed and summarized content along four categories: knowledge of and attitudes towards human rights and human rights education; links between human rights education and social studies; opportunities for adapting the textbook; and obstacles for its adaptation.

### Limitations of the research

This research involved only one case-study textbook and thus the results are not generalizable for all social studies textbooks. However, this case study does provide insights regarding the treatment of human rights that is likely to be relevant for other textbook users, authors, and publishers interested in integrating human rights into learning materials and practices.

## Results

Here, I summarize the results of the quantitative and qualitative analyses of the textbook, and the analysis of the author interviews.

### Textbook analysis

#### Extent to which human rights featured in the textbook

Human rights did not feature prominently in the textbook. There were only a few occurrences of the word “human rights”. No paragraph or extended text was dedicated to explaining human rights. Although one chapter was titled “The Human Rights Dilemma”, the treatment of human rights was reduced to “fundamental freedoms” that were not presented as rights but as values. Value dilemmas were the main thread in the textbook. Thus, when reference to human rights concepts did occur, it was indirect and through the treatment of values. This explained 95% of the incidences when human rights concepts were identified in the textbook. Below are some examples:

- The core value freedom consists of a number of more concrete values: freedom of expression, freedom of religion, […] privacy, freedom of association, freedom of assembly. (p. 99)
- Some people think it is important that everybody is treated equally and that nobody may be insulted (equality). Others on the contrary think that you should be able to say what you want and plead for freedom of expression. This is a clash of values. (p. 19)

However, the textbook did not explain or discuss these freedoms and dilemmas from a human rights perspective, meaning it did not link them with the human rights principles of universality, respect, and non-discrimination.

#### Correctness of content

I performed the content analysis only on the basis of the selected criteria of human rights education. The textbook’s most notable factual error was its reference to the European Convention on Human Rights instead of European Union (EU) treaties, when discussing the European Union:

- European Convention on Human Rights in 2000 with **four freedom rights** for European citizens: free movement of goods, services, persons and capital. (p. 260)

Through the lens of human rights education, I found more “errors” when considering possible connotations of phrasing and presentation of concepts that were implicitly linked to human rights. Here, I must stress that human rights education was not an aim of the textbook, and thus the phrasing may be fully justifiable from the actual aims of the book.

The most striking connotations:

- Human rights are values (not rights)
- Human rights are optional/nonbinding (since they are values, not rights)
- Human rights equal freedom rights (what about other rights?)
- The state’s only duty is to respect human rights (there is no duty to protect and fulfil)
- The state can determine that it can violate certain human rights for specific reasons (there are no absolute rights)
- Public order and security are presented as counter values to human rights and presented as an either-or dilemma

Below are sample sentences translated from the textbook to illustrate the above (all emphasis added):

- Among others, thanks to the Dolle Minas [prominent Dutch feminist group in the 1970s that campaigned for equal rights for women], it is now forbidden in the Netherlands **to give** fewer rights to women than to men. (p. 71)
- Homosexuals, heterosexuals, Muslims, non-believers, **should** all have the same rights. (p. 44) (explained in the teacher’s manual as a left-wing opinion)
- Can they **torture** somebody whom they suspect of terrorism in order to get information? (p. 79) (a question in an exercise that does not mention human rights anywhere, not even in the teacher’s manual—let alone the fact that the right not to be tortured is an absolute right)
- **Can public authorities ignore** the constitutional **rights of citizens**, if there is a “special situation”? (p. 226) (example of placing the limitation grounds of certain human rights outside of the human rights framework, and opposing human rights on the basis of the need for public order and security)

Beyond these connotations and misrepresentations regarding human rights content, another important concern was the formulations of certain sentences and their potential personal impact on the learners. Non-discrimination and respect for all children should be the basis of all safe learning environments. The following formulations seem to indicate some privileged bias on the part of the authors, which can negatively affect the inclusion of all children in a classroom.

- After studying the culture dilemma, it’s time to give your own vision of this societal dilemma. How should the Dutch multicultural society be constructed: do you want more uniformity or diversity? . . . If you so wish, you can choose to give your opinion on **how the West should treat Islam**, according to you. (p. 184) (*Question*: Is Islam not long part of the West? How does this question affect Muslim learners in the classroom? How does it affect the non-Muslim learners?)
- In the Netherlands, the culture dilemma has been discussed a lot since the beginning of the 21st century. **How do we deal with Muslims in the Netherlands?** Can pedophiles found their own association? Can Christian schools dismiss homosexual teachers? (p. 83) (Question: Who is ‘we’ according to this book? How does this formulation affect Muslim learners in the classroom? How does it affect the non-Muslim learners?)

#### Important omissions and missed opportunities

Key human rights content was omitted from the textbook. This section overviews these key omissions according to the main categories of the analytical framework and the implications for learners’ deficits in gaining human rights knowledge.

Regarding *human rights in general*, these were not explained anywhere as universal, inalienable rights based on the values of freedom, equality and respect. The textbook did not mention that human rights have been inscribed for the most part into binding treaties which oblige states parties to respect, protect and fulfil these rights. Consequently, the book did not mention that states have a large margin of discretion when ensuring the realization of their duties and fulfilment of these rights (which would link nicely to the political and social dimension of social studies). Except for the Universal Declaration of Human Rights, the book mentions no treaty by name or reference, let alone explains the treaty obligations. Most striking was the absence of the European Convention on Human Rights, especially since it is directly applicable to the Netherlands and can be invoked in national and international courts.

Regarding *protection mechanisms*, the Dutch Constitution featured relatively prominently, but the textbook did not link the constitutional rights it mentioned to human rights. Consequently, the Constitution was not presented as a protection mechanism of human rights. The book also did not make explicit the relationship between democracy and rule of law on the one hand, and human rights on the other. Further, the textbook did not explicitly name other national protection mechanisms such as the Netherlands Institute for Human Rights (the Dutch independent national human rights institution— in Dutch, *College voor de Rechten van de Mens*); nor did it anywhere describe the role and function of the European Court of Human Rights or the Court of Justice of the EU. The textbook also did not address international protection mechanisms such as the treaty bodies, the UN Security Council, the International Criminal Court, or the various UN agencies that oversee the realization of specific human rights. It also did not discuss the role NGOs can play in the protection of human rights.

As regards *specific human rights*, the textbook did not explain the different groups of rights. In the chapter about the rule of law, it made a distinction between civil rights, political rights, and social rights, but the explanation did not cover their meaning according to the human rights framework. It never touched on the concepts of economic and cultural rights. Moreover, as indicated earlier, while the book does discuss different individual rights implicitly—and sometimes explicitly—it never presents them as rights, only as values. Thus, the textbook approach does not support learners’ recognizing rights as inalienable human rights that they and other people have simply because they are human, as inscribed into respective international human rights treaties. Further, it also underrepresents the important principles of non-discrimination within specific rights and the interrelatedness between various rights.

When looking at the category *“human rights in practice”,* the book fails to link the history of human rights to human rights. Although it looks at certain parts of the history of human rights (such as the French Revolution and the U.S. Declaration of Independence), it does not link these to the development of human rights. In particular, it does not mention the horror of the Second World War as a reason for developing the Universal Declaration. It does not explain the universality of human rights or the various forms of criticism on the universality of human rights. At the same time, its presentation of (certain) human rights as “mere” values that one may or may not adhere to might imply that these are not rights at all. Rather, dilemmas and situations of clashing rights (presented as opposing values) did feature quite heavily in the textbook, but, again, the book did not present them from a human rights perspective. Moreover, the textbook did not state that some rights, such as the right not to be tortured, are absolute. Similarly, when it described situations which were clearly human rights violations it failed to explicitly describe them as such. The authors note “conflicting values” such as freedom of religion and freedom of expression, but they missed the opportunity to explain the principles of human rights and how to find a just balance when rights seem to conflict. This was equally so in terms of the various exercises with sources that mention human rights violations (in other countries)—but that are not explained as such.

As concrete examples of missed opportunities one could consider (all emphasis added):

- In the ideal society of the extreme-right the ‘weak’ are to serve the strong: “**inferior persons”** are **enslaved**, or **banished** to another area/ country of **killed.** (p. 47) (without reference to prohibition of genocide or prohibition of slavery)
- There was no place for Jews, homosexuals and gypsies and they were **removed from the country through a violent struggle**. (p. 30) (description of Nazism and fascism during the Second World War; moreover: is this not downplaying the Holocaust?)

### Author interviews

The textbook analysis revealed key omissions and misconceptions regarding human rights, such as the importance of freedom rights over other rights and the absence of any kind of information on the binding nature of human rights. Therefore, it was important to apprehend the textbook authors’ knowledge and understanding of human rights and to consider any bearing that this might have had on their representation of human rights in the social studies textbook. Following the textbook analysis, I interviewed the authors of the book to assess their knowledge of and attitudes towards human rights and human rights education. The (collective) academic backgrounds of the authors included political science, social science, and social studies teacher-training. Only one of the authors was familiar with the approaches of (global) citizenship education, peace education, and human rights education.

Below, I paraphrase the results of the open-ended questions, with the answers of all three interviewees combined. On the topic of *human rights education,* the authors had various ideas:

- Human rights education is largely about knowledge: that you know that you and others as humans have rights.
- Human rights education means that learners learn which fundamental rights people have and that there are different opinions about that.
- Human rights education is about different human rights, the groups and history of human rights, but also about values education. To be able to do human rights education, you need political knowledge. That includes transmitting knowledge about political struggles, interests, and values through the pedagogy of the social sciences.

To some extent, the authors did have an understanding of what human rights education is and how it could be linked to social studies. Regarding *knowledge of human rights* as described in the table above, I found the following interpretations:

- Social rights are less important than civil and political rights.
- The (human) rights most relevant to the Dutch learner are freedom rights, and more specifically those in the Dutch Constitution. Other human rights, such as the right to shelter, food, or protection, may exist, but are irrelevant for the Netherlands. They may be relevant in situations of war and for people in less fortunate situations than those in the West.
- Human rights are subject to debate. The authors referred to the Universal Declaration of Human Rights but mentioned that this is a nonbinding declaration, on which opinions differ. As an example of that they mentioned the Islamic Declaration of Human Rights.

The findings from the content analysis of the authors’ subjective theory of human rights seemed to coincide with those of the textbook content analysis; that is, the authors had an understanding of human rights as values, not binding rights, and a bias towards freedom rights (values).

In response to the *opportunities and challenges for adaptation of the textbook,* the authors indicated a number of practical concerns for not including more treatment of human rights in the textbook. These include the fact that human rights did not feature in the current curriculum, the limited study of social studies (mandatory only for 60 hours total in the learner’s school career, usually spread over 1 or 2 years), a conscious choice to reduce “facts and figures” and focus on skills, commercial concerns, and the opinion that human rights education goes against their pedagogical belief in value-neutral education. The authors consistently emphasized the last: their aim was to communicate and explain various (opposing) values through dialogue, not to transfer values to the learners. They believed that learners would come to understand and develop their own values through dialogue and did not want to impose their views or imply which values are “right” or “wrong”, which they believed human rights education would.

## Conclusion

This research focused on an investigation of the extent to which human rights featured in the school subject of social studies in the Netherlands. I based this research on a case study of one of the textbooks that social studies teachers use in upper secondary school.

My research showed that human rights feature very little in the textbook. This is despite the fact that (1) international and national policy documents and academic literature link citizenship education and human rights education, and (2) the subject of social studies in the Netherlands has citizenship education as one of its main functions.

These findings are in line with earlier textbook analyses in the area of human rights education, which conclude that most school textbooks only feature human rights in an implicit way (Druba 2006; Weinbrenner and Fritzsche 1998). Recent research and textbook analyses from the Netherlands equally conclude that human rights only feature implicitly in most books (Oomen and Vrolijk 2010; SLO 2012). An extensive schoolbook analysis (Druba 2006) of 95 textbooks from neighboring Germany concludes that it is not unusual for a textbook to only mention the Universal Declaration of Human Rights as a nonbinding declaration, and not to pay attention to the subsequent legally binding international human rights conventions. Textbooks also rarely mention more recent treaties, such as the Convention on the Elimination of All Forms of Racial Discrimination. In Germany, as well, most schoolbooks emphasize values education without linking the moral and legal aspects of human rights. Moreover, none of the exercises in the analyzed German schoolbooks requires learners to use human rights as a tool to judge political relations, whether in domestic affairs or foreign-policy measures.

The results of this case study are to some degree understandable. First, human rights education was not a goal of the authors who wrote the textbook. Their point of departure was the exam program and citizenship education from the perspective of the pedagogy of values education. Human rights was not seen as central to any of these concerns. Second, the authors were not experts on human rights. Pingel (2010) underlined that textbook authors cannot be experts in all subjects they describe in a schoolbook. This can explain, to a large degree, the omissions in the area of human rights as well as the questionable connotations certain formulations elicited when seen through a human rights lens. Third, as the authors explained, they had not only pedagogical considerations but also practical and pragmatic concerns when developing the book. Such concerns can override content considerations.

Nevertheless, we can also say that the case study reveals opportunities where authors may easily incorporate human rights content and themes. Thus, educators can—and should—integrate human rights education into the subject of social studies in the Netherlands. Citizenship education is incomplete without certain elements of human rights education. Whether or not a person subscribes to human rights (In the context of a “devaluation” of, and pressure on, human rights in the Netherlands and Europe, see, e.g., Amnesty International Netherlands 2017, O’Flaherty 2017 and Silvis 2016), the Netherlands has bound itself to legally binding human rights treaties, which citizens can claim through the rule of law. Equally, democracy relies heavily on respect for various civil and political human rights—such as the freedom of expression, the right to vote, and the freedom of association—but equally on socioeconomic rights such as the right to education. Thus, teaching about human rights does not necessarily interfere with value neutrality, as human rights are part of international and national law, and of democracy and the rule of law.

At the same time, human rights education is not complete without political and social literacy, as well as political and social judgment. What are the underlying values of human rights, how do different countries and political parties interpret rights and limitations, how do we (as citizens in our societies) ensure that human rights are respected and fulfilled—through which laws and policies—and why are human rights not always respected, protected, or realized in practice? These are examples of questions that the infusion of human rights within citizenship education might help to address.
